# Nephrometry score-guided off-clamp laparoscopic partial nephrectomy: patient selection and short-time functional results

**DOI:** 10.1186/s12957-016-0914-5

**Published:** 2016-06-21

**Authors:** Hong-Kai Wang, Xiao-Jian Qin, Chun-Guang Ma, Guo-Hai Shi, Hai-Liang Zhang, Ding-Wei Ye

**Affiliations:** Department of Urology, Fudan University Shanghai Cancer Center, 270 Dong’an Rd (M), Shanghai, 200032 China; Department of Oncology, Shanghai Medical College, Fudan University, Shanghai, People’s Republic of China

**Keywords:** Renal cell carcinoma, Laparoscopic partial nephrectomy, Zero ischemia, Nephrometry score

## Abstract

**Background:**

Laparoscopic partial nephrectomy (LPN) is not a novel but a relatively technically challenging surgical procedure. Off-clamp LPN with zero ischemia can completely eliminate ischemic reperfusion injury to the kidney. The purpose of this study was to evaluate the safety and functional outcome of nephrometry score-guided off-clamp technique in LPN.

**Methods:**

A total of 44 patients underwent LPN between January 2015 and July 2015 for renal mass with radius, exophytic/endophytic, nearness to sinus, anterior/posterior location (RENAL) score 4 were enrolled. Twenty-two of them underwent off-clamp LPN with zero ischemia, and the other 22 received standard LPN with common renal artery clamp. Estimate blood loss (EBL), total operation time, resection time, renorrhaphy time, preoperative estimated glomerular filtration rate (eGFR), postoperative eGFR, eGFR change, and drainage after surgery were compared between these two groups using *t* test.

**Results:**

Patients’ characteristics including gender, age, BMI, tumor size, and RENAL score were balanced between the two groups. Average EBL was more in the off-clamp group than in the on-clamp group (134.32 versus 70.23 ml, *p* = 0.001). Average eGFR change was less in the off-clamp group than in the on-clamp group (−1.56 versus −6.45, *p* < 0.001). Average drainage after surgery was 203.41 ml for the off-clamp group and 145.46 ml for the on-clamp group, *p* = 0.062. No urinary leakage and hematuria occurred in both groups. There were no statistical difference in total operation time, resection time, renorrhaphy time, preoperative eGFR, and postoperative eGFR between the two groups.

**Conclusions:**

Off-clamp LPN is a safe and feasible approach to excise certain kidney tumors with RENAL score 4. This technique can better preserve kidney function without ischemic reperfusion injury.

## Background

The incidence of renal cell carcinoma (RCC) has been increasing over the past decades [1, 2]. Radical nephrectomy (RN) remains the standard care for localized tumors, while this surgery procedure largely decreases kidney function and sequentially exposes the patient to greater cardiovascular morbidity and mortality risks in their future time [3]. According to the 2015 NCCN guideline, partial nephrectomy (PN) is recommended to preserve kidney function for patients with an AJCC stage T1a tumor. However, ischemic reperfusion injury is inevitable if renal artery is temporary clamped during the surgical procedure [4]. A recent study indicated that every minute of ischemia imparted additional risk for postoperative renal dysfunction [5]. Cold ischemia method may theoretically provide better protection to kidney function, while the benefit is minimal because it usually prolongs the ischemic time for cooling the kidney [6].

The term “zero ischemia” implies that both tumor resection and renorrhaphy were completed without hilar clamping and ischemic stress [7]. Selective/segmental renal artery clamping is used to preserve the remnant kidney tissue besides the tumor [8, 9]. Nevertheless, it is not real zero ischemia for the whole kidney, and only skillful oncological urologist can perform this challenging work [10]. Laser-supported [11], radio frequency (RF)-assisted [12], hydro-jet-assisted [13–15], and microwave-assisted [16] off-clamping PN can achieve true zero ischemic surgery. However, positive surgical margin or complications such as calyceal injury, urinary leakage, and venous injuries are difficult to completely eliminate in these ablation assisted PN [17, 18].

Anatomy-based nephrometry scoring systems such as radius, exophytic/endophytic, nearness to sinus, anterior/posterior location (RENAL) score [19], preoperative aspects and dimensions used for anatomic (PADUA) classification [20], centrality index [21], and contact surface area [22] have been assigned to guide clinical decisions on nephron-sparing surgery or radical nephrectomy.

We here adopted a novel RENAL score-guided off-clamp laparoscopic partial nephrectomy (LPN) technique in selected patients, to completely avoid renal ischemic injury and prevent some of the incidents related to renal hilar dissection and clamping during LPN. In this paper, we present our experience with off-clamp LPN and demonstrate the patient selection criteria and the outcome of this technique compared with standard on-clamp LPN.

## Methods

### Patients

After institutional review board approval, we enrolled 44 consecutive patients who underwent retroperitoneal LPN at our institute, between January 2015 and July 2015. The including criteria on kidney tumors were exophytic, solid or cystic, RENAL score 4 [19]; maximum diameter ≤3 cm; and suspicious for malignancy on computed tomography (CT) scan or magnetic resonance image (MRI). All the LPNs were performed sequentially one by one for off-clamp LPN with zero ischemia or standard LPN with common renal artery clamp by the same surgeon (Ye DW). Twenty-two patients underwent off-clamp LPN, and the other 22 received standard LPN. Patients’ demographics; main operative and outcome variables including estimate blood loss (EBL), total operation time, resection time, renorrhaphy time, preoperative estimated glomerular filtration rate (eGFR), 24–48-h postoperative eGFR, eGFR change, drainage after surgery, and length of hospital stay; and tumors’ histopathological results (size, site, side, and grade) were prospectively collected according to Fuhrman grading system and the 2004 WHO classification [23].

### Major surgical procedures

**Fig. 1:**
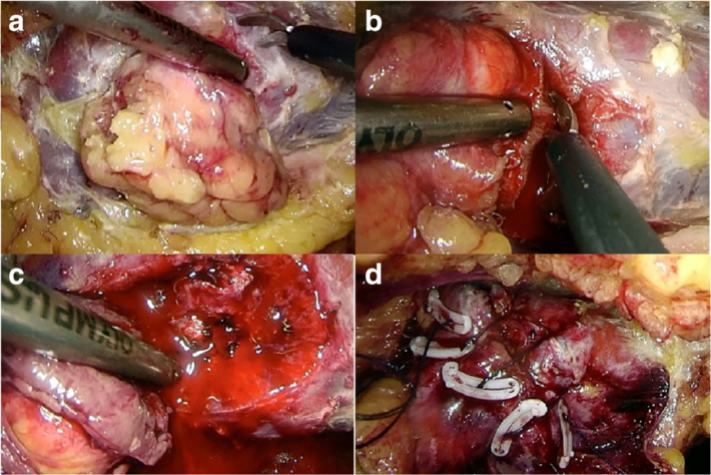
Surgical procedure for off-clamp laparoscopic partial nephrectomy. **a** Mobilization of the kidney and complete exposure of the tumor. **b, c** Tumor resection using cold scissors. **d** Renorrhaphy using Hem-o-lok clips to tighten and secure the sutures at each exit point

The patients were administered general anesthesia and placed in the lateral decubitus position. Three ports in the lumbar region were applied. After establishing the retroperitoneal cavity, the kidney was mobilized as necessary to expose the tumor completely (Fig. 1a). Intraoperative ultrasound was used to measure the diameter and depth of the tumor. Before resection of the tumor, the common renal artery was dissected and clamped only in the 22 patients under on-clamp LPN. Scissors were used to open the renal capsule 2~3 mm away from the tumor and further cutting deep into the renal cortex slowly and carefully around the tumor (Fig. 1b, c). Bipolar coagulation was applied when small arterial bleeding occurred. After complete excision of the tumor, the margin of resection was sent to pathology for the frozen section to ensure complete excision of the tumor. Then, the parenchymal defect was closed using Hem-o-lok clips to tighten and secure the sutures at each exit point (Fig. 1d). Pneumoperitoneum pressure was temporarily risen to 18 mmHg during tumor resection and renorrhaphy. No hemostatic agents were used during the procedure.

### Follow-up

Preoperative serum creatinines were performed within 1 week before surgery, and postoperative measurements were tested within 1 week after surgery. Estimated GFR (eGFR) (units = ml/min/1.73 m^2^) was calculated by the modification of diet in renal disease equation [24].

### Statistical analyses

All statistical analyses were performed using SPSS version 19.0 statistical software (SPSS Inc., Chicago, IL) after conducting the univariate analyses for the variable, the relationship between the main studied variables, serum creatinine values, EBL, length of hospital stay, main surgical complications, patients’ age and gender, and subtype, size, and grade of tumors. Categorical data were analyzed using chi-square. Pearson correlation coefficients were calculated between continuous variables. *p* values of 0.05 or less were considered statistically significant.

## Results

### Preoperative patient and tumor demographics

A total of 44 patients (22 off-clamp LPN, 22 on-clamp LPN) participated in the study (Table 1). The mean age was 54.36 years for the off-clamp group and 54.41 years for the on-clamp group. Male to female ratios were both 16:6 for the two groups. BMI was equal between groups, mean 25.43 versus 24.10 kg/m^2^. Mean tumor size and intra-kidney tumor size (maximum tumor diameter within renal parenchyma) were 19.86 and 18.46 mm for the off-clamp group and 19.14 and 17.68 mm for the on-clamp group, respectively, also having no statistical difference. There was also no statistical difference between two groups in tumor side and position.

**Table 1:** Patients’ preoperative demographics

Factors	Off-clamp	On-clamp	*p* value
	(22 cases)	(22 cases)	
Gender			
Male	16	16	1.000
Female	6	6	
Age (years, mean ± SD)	54.36 ± 12.07	54.41 ± 10.40	0.989
Height (cm, mean ± SD)	166.68 ± 6.59	166.09 ± 7.49	0.782
Weight (kg, mean ± SD)	71.00 ± 11.77	66.52 ± 9.01	0.164
BMI (kg/cm^2^, mean ± SD)	25.43 ± 2.83	24.10 ± 2.76	0.122
Tumor size (mm, mean ± SD)	19.86 ± 5.48	19.14 ± 5.97	0.676
Tumor size intra-kidney (mm, mean ± SD)	18.46 ± 4.13	17.68 ± 4.69	0.565
Side of the kidney			
Left	12	7	0.128
Right	10	15	
Position of tumor			
Upper pole	5	4	0.831
Middle	10	12	
Lower pole	7	6	

### Surgical outcome

All patients had negative surgical margins without complications of postoperative hematuria, delayed bleeding, and urinary leakage. There was no need to convert any of the cases to clamped technique or to the open technique. Average EBL was 134.32 ± 70.11 and 70.23 ± 39.75 ml in the off-clamp group and the on-clamp group, respectively, *p* < 0.001. Tumor resection time was longer for the off-clamp patients than for the on-clamp patients (5.86 ± 2.17 versus 4.55 ± 1.47 min, *p* = 0.023). No statistical difference was detected between the two groups on total operation time, renorrhaphy time, postoperative drainage, postoperative bed time, and main surgical complications (Table 2).

**Table 2:** Postoperative characteristics

Factors	Off-clamp	On-clamp	*p* value
	(22 cases)	(22 cases)	
Subtypes			
Clear cell	16	16	0.446
Papillary	4	2	
Chromophobe	2	2	
Oncocytoma	0	2	
Grade			
1	2	2	0.462
2	14	9	
3	4	7	
Not applicable	2	4	
Resection time (min, mean ± SD)	5.86 ± 2.17	4.55 ± 1.47	0.023
Renorrhaphy time (min, mean ± SD)	16.00 ± 3.75	14.73 ± 2.25	0.18
Estimated blood loss (ml, mean ± SD)	134.32 ± 70.11	70.23 ± 39.75	0.001
Drainage (ml, mean ± SD)	203.41 ± 124.17	145.46 ± 68.29	0.062
Operation time (min, mean ± SD)	75.00 ± 16.97	82.27 ± 16.10	0.152
Postoperative bed time (day, mean ± SD)	5.77 ± 1.19	5.68 ± 1.13	0.796
eGFR preoperation (ml/min/1.73 m^2^, mean ± SD)	86.43 ± 19.92	90.66 ± 21.13	0.594
eGFR postoperation (ml/min/1.73 m^2^, mean ± SD)	84.87 ± 21.72	84.21 ± 28.77	0.932
eGFR change (ml/min/1.73 m^2^, mean ± SD)	−1.56 ± 4.70	−6.45 ± 3.83	<0.001
Hematuria	0	0	1.000
Postoperative hemorrhage	0	0	1.000
Blood transfusion	0	0	1.000
Urinary leakage	0	0	1.000
Clavien-Dindo classification of surgical complications			
Grades 1–2	22	22	1.000
Grades 3–5	0	0	

### Pathological outcomes

Histopathological subtypes were clear-cell RCC (*n* = 32, 16 in the off-clamp group and 16 in the on-clamp group), papillary RCC (*n* = 6, 4 in the off-clamp group and 2 in the on-clamp group), chromophobe RCC (*n* = 4, 2 in the off-clamp group and 2 in the on-clamp group), and oncocytoma (*n* = 2, 2 in the on-clamp group). Fuhrman grades were grade 1 (*n* = 4, 2 in the off-clamp group and 2 in the on-clamp group), grade 2 (*n* = 23, 14 in the off-clamp group and 9 in the on-clamp group), grade 3 (*n* = 11, 4 in the off-clamp group and 7 in the on-clamp group), and not applicable (*n* = 6, 2 in the off-clamp group and 4 in the on-clamp group).

### Renal functional evaluation

Mean preoperative serum eGFRs were 86.43 ± 19.92 ml/min in the off-clamp group and 90.66 ± 21.13 ml/min in the on-clamp group, *p* = 0.594. Mean postoperative serum eGFRs were 84.87 ± 21.72 ml/min in the off-clamp group and 84.21 ± 28.77 ml/min in the on-clamp group, *p* = 0.932. Mean eGFR changes were −1.56 ± 4.70 ml/min in the off-clamp group and −6.45 ± 3.83 ml/min in the on-clamp group, *p* < 0.001.

## Discussion

Minimally invasive nephron-sparing surgery has become a favorable option by many surgeons and many patients as it is showing outstanding oncologic outcome and at the same time maintaining good renal function [25, 26]. How to better preserve the renal function has always been a key problem in the treatment of kidney tumors. A few auxiliary means, such as cold ischemia, selective renal artery clamping, segmental renal artery clamping, renal parenchymal clamping, superselective embolization, radio frequency, laser, microwave, and hydro-jet, have achieved some benefits, while these methods have their own disadvantages and deficiencies [27]. We here proposed the RENAL score guidance for the retroperitoneal laparoscopic zero ischemia nephron-sparing surgery and performed tentative attempts to obtain preliminary outcomes.

The existing nephrometry scoring systems can assist in clinical decision-making on radical nephrectomy (RN) versus PN or open versus minimally invasive PN. They can also inform the surgeon regarding technical difficulty during minimally invasive PN for a given mass and have been correlated with ischemia time, operation time, blood loss, complications, and the likelihood of conversion from PN to RN [28]. However, they lack evidence in guiding off-clamp zero ischemia minimally invasive PN. We first introduced RENAL score to guide the zero ischemia LPN, and the results show that this surgical technique was safe and feasible in RENAL score 4 renal mass.

The RENAL nephrometry score consists of five anatomic radiologic properties: (R)adius/maximal tumor diameter, (E)xophytic/endophytic properties, (N)earness to the collecting system or sinus, (A)nterior(a)/posterior(p)/not anterior or posterior (x) descriptor, and (L)ocation relative to the polar line. For each variable except A, 1 to 3 points are assigned, which yield a total of 4 points for the least complex and 12 points for the most complex mass. Masses are classified as low complexity (RENAL scores 4–6), moderate complexity (scores 7–9), or high complexity (scores 10–12) [19]. Herein, we choose tumors of RENAL score 4 and maximum diameter less than 3 cm as candidates for off-clamp retroperitoneal LPN, because these tumors are the least complex with least surgical complications including bleeding, renal pelvis injury, urinary leakage, and hematuria [29]. Thompson et al. also proposed that off-clamp PN can reduce the hazard of both acute and chronic kidney disease. Our outcomes demonstrated that off-clamp operation was safe in these well-selected cases, with only a bit more bleeding but better protection of kidney function than that of the on-clamp group.

EBL was more in the off-clamp patients than in the on-clamp cases. This was reasonable for zero ischemia surgery. We temporarily raise pneumoperitoneum pressure to 18 mmHg when removing the tumor and suturing the wound of the kidney. We hope this procedure can decrease blood loss during off-clamp LPN. Rizkala and colleagues had described their novel zero ischemia robotic PN technique in 2013, that is, sequential preplaced suture renorrhaphy technique [30]. Compared to straightforward excision without hilar clamping, preplacing sutures sequentially aids in providing better visualization secondary to a decrease in bleeding onto the tumor bed. However, this procedure is completed under robotic surgery, and we may attempt to add this technique to our zero ischemia LPN in the future.

Postoperative drainage also seemed to be more in the off-clamp group, although there was no statistical significance. We used cold scissors and suction to remove the tumor because this could provide better incision plane to ensure complete resection and not cut into the tumor in such a continuous bleeding situation. Energy-cutting equipment such as Valley ForceTriad energy platform, high-frequency electrosurgical equipment, and a HARMONIC ACE+ shears ultrasonic knife were not considered suitable for tumor resection in an off-clamp zero ischemic situation with continuous bleeding. Coagulation was poor with a lot of eschar which made the plane blurred and indistinct for perfect resection. ERBE VIO + BiSect/BiCamp bipolar coagulation was effective in handling small artery hemorrhage but difficult in controlling a large area of venous bleeding from the tumor bed. For venous bleeding, suturing remains the most reliable method.

The kidney tissue was more brittle in off-clamp condition and easy to be torn during suturing if the suture was pulled too hard [28]. We preferred to use Polysorb-braided absorbable suture 1 (COVIDIEN Inc.) in renorrhaphy, because it was thicker enough to reduce cutting into normal kidney tissue and firm enough to pull the apart kidney incision together. Barbed sutures are not recommended because they are too rough and bleeding is more prone to occur in the needle site.

A limitation of our technique is its limited use for high RENAL score tumors. For those tumors, robotic surgery may be helpful in better tumor resection and renorrhaphy. Another limitation for the current study is its small sample size with only short-term results. External validation of these data with larger cohorts and long-term follow-up especially eGFR change over 12 months are required.

## Conclusions

We compared off-clamping and main artery clamping in LPN. The off-clamping cohort was associated with a longer tumor resection time and more EBL. Functional outcome of eGFR changes seemed superior in the off-clamping cohort. As we continue to implement this technique, we hope to further assess its long-term safety and oncological effectiveness.

## Abbreviations

LPN: Laparoscopic partial nephrectomy
EBL: Estimate blood loss
eGFR: Estimated glomerular filtration rate
BMI: Body mass index
RCC: Renal cell carcinoma
RN: Radical nephrectomy
PN: Partial nephrectomy
NCCN: National Comprehensive Cancer Network
AJCC: American Joint Committee on Cancer
CT: Computed tomography
MRI: Magnetic resonance image
